# How to Choose the Right Inducible Gene Expression System for Mammalian Studies?

**DOI:** 10.3390/cells8080796

**Published:** 2019-07-30

**Authors:** Tuula Kallunki, Marin Barisic, Marja Jäättelä, Bin Liu

**Affiliations:** 1 Cell Death and Metabolism, Center for Autophagy, Recycling and Disease, Danish Cancer Society Research Center, 2100 Copenhagen, Denmark; 2 Department of Drug Design and Pharmacology, Faculty of Health Sciences, University of Copenhagen, 2200 Copenhagen, Denmark; 3 Cell Division and Cytoskeleton, Danish Cancer Society Research Center, 2100 Copenhagen, Denmark; 4 Department of Cellular and Molecular Medicine, Faculty of Health Sciences, University of Copenhagen, 2200 Copenhagen, Denmark

**Keywords:** tetracycline, cumate, light-switchable, tamoxifen

## Abstract

Inducible gene expression systems are favored over stable expression systems in a wide variety of basic and applied research areas, including functional genomics, gene therapy, tissue engineering, biopharmaceutical protein production and drug discovery. This is because they are mostly reversible and thus more flexible to use. Furthermore, compared to constitutive expression, they generally exhibit a higher efficiency and have fewer side effects, such as cell death and delayed growth or development. Empowered by decades of development of inducible gene expression systems, researchers can now efficiently activate or suppress any gene, temporarily and quantitively at will, depending on experimental requirements and designs. Here, we review a number of most commonly used mammalian inducible expression systems and provide basic standards and criteria for the selection of the most suitable one.

## 1. Introduction

Classic genetic studies are based on correlating genetic alterations with the resulting phenotypes. Several important signaling pathways, including mTOR [1,2], apoptosis [3], autophagy [4] and Hippo [5,6] pathways, have been discovered by classic genetics. Given the fact that some lethality-causing genes or essential genes are impossible to overexpress or knock out, fine-tuning their expression is necessary for the analysis of their function [7]. Moreover, the irreversible manipulation of gene expression often drives compensatory adaptation in higher organisms [8,9]. Therefore, the ability to switch the gene expression on and off or to modulate the level of gene expression in a quantitative and temporal way can preferentially reveal the direct consequence of a certain genetic change and provide an additional filter to exclude other side- and off-target effects. This is especially beneficial when working with mammalian cells that are maintained and controlled by highly intricate genetic networks.

Initially, endogenous hormone- or stress-responsive promoters were considered prevalent to achieve temporal induction of gene expression [10,11,12,13,14,15]. This strategy has gradually been vetoed due to their leaky expression and lack of specificity in all physiological conditions. For example, metallothionein promoter that originates from the equine kidney is not only regulated by heavy metals but also by hypoxia, oxidative stress, and hormones, making it inappropriate for studies involving these biological processes [16,17,18]. To overcome these drawbacks, discoveries of bacterial operons have inspired scientists to transfer these prokaryotic genetic elements into mammalian cells. The first successful attempt with prokaryotic operons was made in 1987, when the *Escherichia coli* Lac operator-repressor (LacR/O) system was used to switch on gene expression by adding isopropyl β-D-thiogalactopyranoside (IPTG) into mouse cells [19]. Since then, tremendous efforts have been made to develop many advanced inducible systems, which have been exploited to overexpress, knock down and regulate, knocking out specific genes temporarily. These developments and recent progress in the field are discussed below.

## 2. Tetracycline-Controlled Operator System

### 2.1. Induction of Target Gene

The above-described lacR/O-based systems were soon found to be too limited due to their inefficiency and moderate potency in mammalian cells. Even though a chimeric lacR-VP16 has been described to activate a minimal promoter almost 1000-fold at elevated temperatures in the presence of IPTG [20], the temperature dependence and the inherent IPTG-related problems were found to limit the usability of this approach.

**Figure 1:**
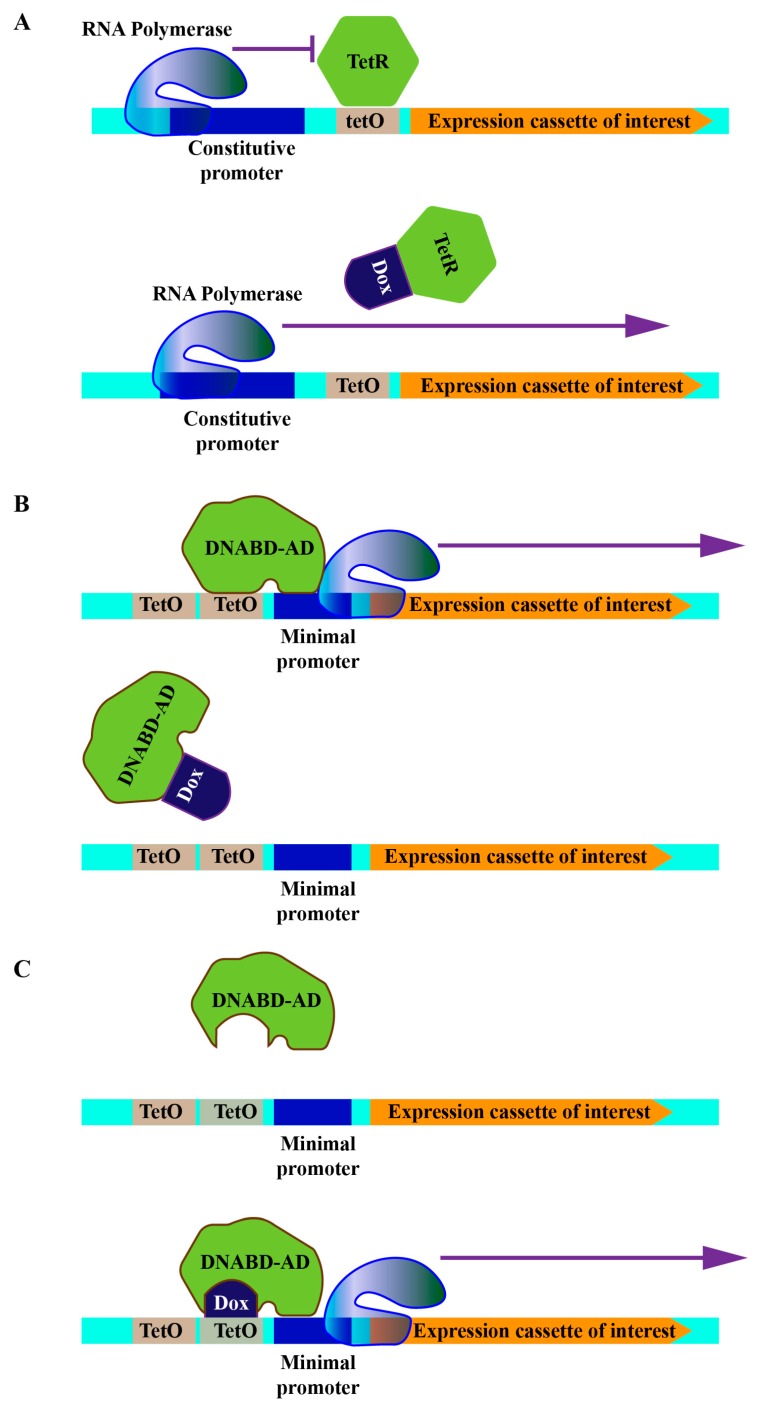
Schematic representations of tetracycline-controled operator systems. (**A**) Repression based configuration. (**B**) Tet-off configuration. (**C**) Tet-on configuration. DNABD: DNA binding domain, AD: activating domain, TetO: tetracycline operator, Dox: doxycycline, TetR: tet repressor.

Soon after, another bacterial regulatory element, the Tn10-specified tetracycline-resistance operon of *E. coli*, was found to exhibit a superior performance and became a popular tool to control mammalian gene expression [21,22]. Currently, there are three configurations of this system: (1) The repression-based configuration, in which a Tet operator (TetO) is inserted between the constitutive promoter and gene of interest and where the binding of the tet repressor (TetR) to the operator suppresses downstream gene expression. In this system, the addition of tetracycline results in the disruption of the association between TetR and TetO, thereby triggering TetO-dependent gene expression (Figure 1A). (2) Tet-off configuration, where tandem TetO sequences are positioned upstream of the minimal constitutive promoter followed by cDNA of gene of interest. Here, a chimeric protein consisting of TetR and VP16 (tTA), a eukaryotic transactivator derived from herpes simplex virus type 1, is converted into a transcriptional activator, and the expression plasmid is transfected together with the operator plasmid. Thus, culturing cells with tetracycline switches off the exogenous gene expression, while removing tetracycline switches it on (Figure 1B). (3) Tet-on configuration, where the exogenous gene is expressed when tetracycline is added to the growth medium. Even though tetracycline is nontoxic to mammalian cells at the low concentration required to regulate TetO-dependent gene expression, its continuous presence is suboptimal in a variety of experimental setups. Moreover, the regulation is usually slow when the effector has to be removed by multiple washes [23]. Thus, a mutant tTA with four amino acid substitutions, termed rtTA, was developed by random mutagenesis of tTA [24,25]. Unlike tTA, rtTA binds to TetO sequences in the presence of tetracycline, thereby activating the silent minimal promoter (Figure 1C).

Based on the three configurations described above, several additional optimizations have been made. One of these was an attempt to further reduce the leakage of the system. In the repression-based configuration, transcriptional repressor domains, such as the Krüppel-associated box (KRAB) of human KOX1 [26,27], have been fused with tetR to reduce the leakage. In the Tet-on configuration, newly engineered rtTAs with few mutations make them exponentially active and sensitive. More importantly, these rtTA variants show no activity in the absence of doxycycline (a synthetic tetracycline derivative) [28]. Recently, another mutation, TetRI194T, on top of the above rtTAs, was shown to have an even more superior performance [29]. However, there is still a major drawback associated with the tetracycline-induced operator system. That is that upon continuous cell culture, some cell lines can spontaneously lose their inducibility, especially after successive selection rounds [30]. Finally, it should also be noted that tetracycline-derived contaminants that are often present in cell culture serums can cause problems with tetracycline-based expression systems. These can, however, be avoided using tetracycline-free fetal bovine serum.

### 2.2. Induction of Knockdown or Knockout of Target Gene

Tetracycline-controlled inducible operator systems can also be combined with RNA interference and CRISPR-Cas9 (clustered regularly interspaced short palindromic repeats-CRISPR associated protein 9) to knock down and knock out gene expression, respectively, in mammalian cells. RNA interference (RNAi) has emerged as an essential tool to achieve knockdown of gene expression [31,32]. It employs a small double-strand RNA processed by endoribonuclease DICER to trigger RNA-induced silencing complex (RISC)-dependent mRNA degradation, thereby leading to the subsequent decline of corresponding protein [33]. This can be activated by two means: the delivery of synthetic siRNAs, which induces a transient knockdown of protein expression, or by expressing short hairpin RNA (shRNA), which can be processed by RNAi machinery into siRNA in vivo. Stable transfection of shRNA expressing plasmids into mammalian cells can constitutively knock down specific gene expression [33]. However, in the case where a gene’s knockdown has a deleterious effect on target cells, the inducible expression of shRNA achieved by repression based configuration becomes a more reliable approach [34]. A minor adjustment has to be made to avoid the leaky expression of shRNA driven by RNA-Pol III-dependent promoters (H1 or U6) in the absence of tetracycline, which is two tetracycline operons that need to be placed flanking the TATA box [35,36]. Corresponding lentiviral systems have also been developed for cells that are difficult to transfect [34].

The CRISPR-Cas9 technology has recently revolutionized gene editing. Cleavage of specific DNA site catalyzed by Cas9 endonuclease followed by error-prone non-homologous-end-joint repair can efficiently result in gene knockout [37,38]. Original protocol to generate knockout cells by CRISPR technology requires the selection of positive and negative clones for phenotypic comparison. More than two-three weeks of culturing under selection pressure fosters cells adapted to the loss of the gene of interest. This adaptation may also involve uncontrolled irreversible changes in other genes, if these are advantageous for the survival of the knockout cells. Moreover, the frequently observed clonal variation can make it challenging for researchers to draw reliable conclusions by analyzing the phenotypes of single-cell-derived clones. Thus the inducible expression of Cas9 driven by rtTA can overcome these drawbacks. Comparing non-induced and induced cells within a short time-frame tends to reveal the direct effects caused by the loss of the gene of interest [39,40].

## 3. Cumate-Controlled Operator System

### 3.1. Induction of Target Gene

**Figure 2:**
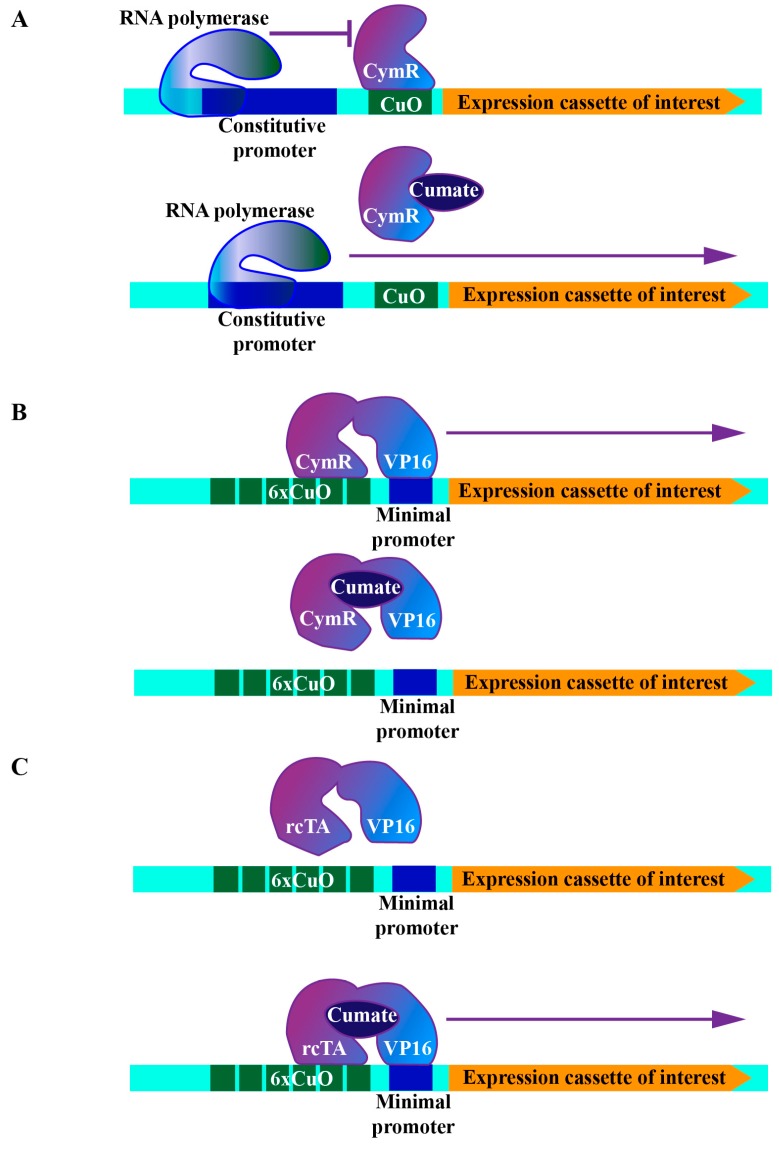
Schematic representations of cumate-controlled operator systems. (**A**) Repression configuration. (**B**) Activator configuration. (**C**) Reverse activator configuration. CymR: cumate repressor, CuO: cumate operator, rcTA: reverse chimeric transactivator.

For particular genetic epistasis analyses, simultaneous or sequential manipulation of the expression of different genes is a fundamental approach. However, such genetic manipulations are hard to achieve in mammalian cells due to the inability to generate genetic cross at will in model animals. Therefore, additional operator systems can be combined with the tetracycline system to establish more complicated genetic setups in mammalian cells (Table 1). Here we introduced the cumate-controlled operator system as an example. This operator originates from the p-cmt and p-cym operons in *Pseudomonas putida*. The corresponding repressor contains an N-terminal DNA-binding domain recognizing the imperfect repeat between the promoter and the beginning of the first gene in the p-cymene degradative pathway [41]. Similarly to a tetracycline-controlled operator system, the cumate operator (CuO) and its repressor (CymR) were engineered into three configurations: (1) The repressor configuration, which is realized by placing CuO downstream of a constitutive promoter, where the binding of CymR to CuO efficiently suppresses downstream gene expression. The addition of cumate releases CymR, thereby triggering downstream gene expression (Figure 2A). (2) Activator configuration, where chimeric molecular (cTA) is formed via the fusion of CymR and VP16. In this configuration, a minimal promoter was placed downstream of the multimerized operator binding sites (6xCuO). Again, gene expression controlled by the minimal promoter was activated by removing cumate from the medium (Figure 2B). (3) Reverse activator configuration, for which after the random mutagenesis and screening, cTA mutant (rcTA) that binds to CuO upon addition of cumate was generated. In this configuration, the addition of cumate triggered downstream gene expression [42] (Figure 2C).

### 3.2. Induction of Knockdown of Target Gene

Cumate-controlled operator system was also further developed for inducible knockdown of gene expression. This system was further developed by System Biosciences (SBI) (Patent NO.: US 8,728,759 B2, US 7,745,592 B2), which provides plasmids and technical instruction as a service. Several successful examples of using this system have been reported, for example a small GTPase, Rheb, which is required for activation of cell growth regulator mTOR1, was characterized by knocking it down by cumate-controlled operator plasmid [43].

## 4. Protein–Protein Interaction-Based Chimeric System

Protein–protein interaction-based chimeric systems provide another strategy to achieve tunable and temporal control of gene expression. This strategy takes advantage of two fundamental observations that have emerged from basic studies of gene expression and signal transduction. It is based on the observation that the DNA-binding domains and activation domains of transcription factors can function independently and retain activity as a heterologous single protein [44,45]. It additionally takes advantage of the fact that many protein–protein interactions can be triggered by chemicals or physical stimuli, such as blue light.

### 4.1. Induction of Target Gene by Control of the Interaction between FKBP12 and mTOR

**Figure 3:**
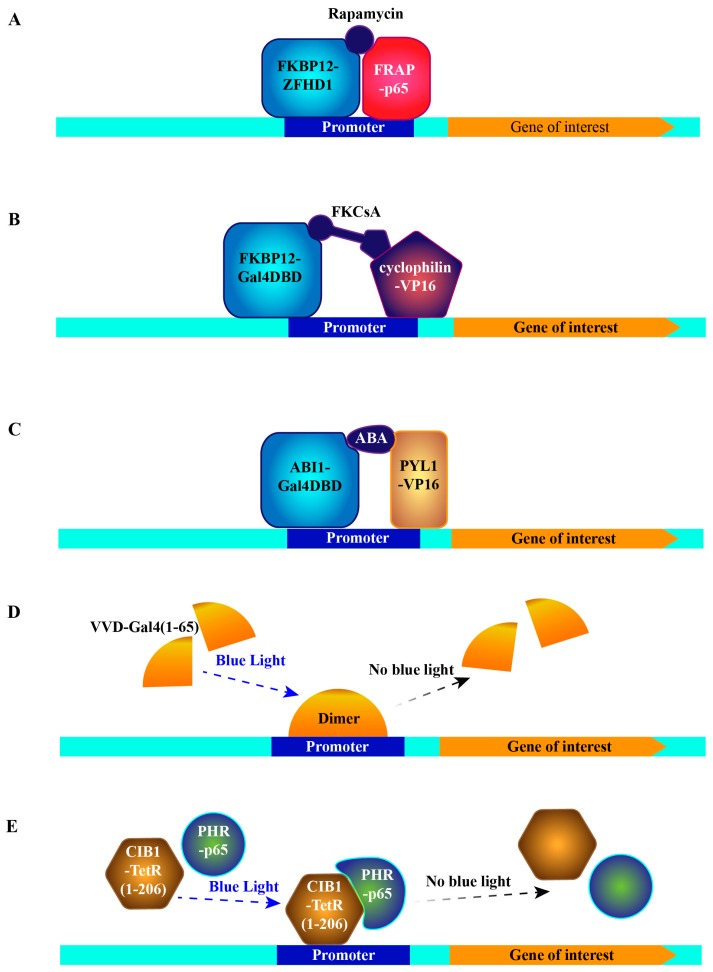
Schematic representations of protein–protein interaction based systems. (**A**) Inducible system dependent on rapamycin induced interaction between FKBP12 and FRAP. (**B**) Inducible system dependent on FKCsA induced interaction between FKBP12 and cyclophilin. (**C**) Inducible system dependent on ABA induced interaction between PYL1 and ABI1. (**D**) Inducible system dependent on blue light induced VVD dimer formation. (**E**) Photoactivatable-Tet-OFF/ON system dependent on blue light induced interaction between Cry2 and CIB1. Gal4DBD: Gal4 DNA binding domain, ABA: abscisic acid, TetR: tet repressor.

The first trial of this concept was made by utilizing the rapamycin-induced interaction between FKBP12 (FK506 binding protein 12) and mTOR [46]. Rapamycin and its analog FK506 bind to a cytosolic protein FKBP12 [47]. This complex further binds to mTOR, forming a tripartite complex [48,49,50]. Therefore, fusing FKBP12 and mTOR with a DNA-binding domain of ZFHD1 [51] and the activation domain of NF-κB p65 protein [52], respectively, bridges both domains to drive expression of the gene of interest in a rapamycin-dependent fashion (Figure 3A) [46]. Due to the immunosuppressive and the cell cycle inhibitory effect of FK506 and rapamycin [48], a new synthetic compound, FKCsA, which is a heterodimer of FK506 and cyclosporin A (an immunosuppressant complexed with protein cyclophilin), was developed and was shown to exhibit neither toxicity nor immunosuppressive effects [53]. To trigger gene expression, the addition of FKCsA to cells hinges FKBP12 fused with the Gal4 DNA-binding domain (Gal4DBD) and cyclophilin fused with VP16, thereby activating expression of the gene of interest downstream of upstream activation sequence (UAS, Gal4DBD binding site) (Figure 3B) [53].

The above-described rapamycin-based systems hold some drawbacks that limit their applications. The major concern is the essential function of endogenous mammalian target of rapamycin complex 1 (mTORC1) in the control of metabolism and growth of mammalian cells [54,55], which can be inhibited by either rapamycin or its analogs. Thus, inhibition or any disturbance of mTORC1 signaling severely changes the metabolic status of cells [56]. An additional problem is the slow kinetics of ceasing the expression after removing rapamycin [57].

### 4.2. Induction of Target Gene by Control of the Interaction between PYL1 and ABI1

Besides the rapamycin system, abscisic acid (ABA)-regulated interaction between two plant proteins was exploited to regulate gene expression in a temporal and quantitive manner in mammalian cells. The two proteins are PYL1 (abscisic acid receptor) and ABI1 (protein phosphatase 2C56), which are important players of the ABA signaling pathway required for stress responses and developmental decisions in plants [58,59]. According to the crystal structure of PYL1-ABA-ABI1 complex [60,61,62], interacting complementary surfaces of PYL1 (amino acids 33 to 209) and ABI1 (amino acids 126 to 423) were chosen for chimeric protein construction. Similarly, Gal4DBD was fused with ABI1 and VP16 with PYL1. Thus after transfecting this ABA-activator cassette and UAS-driven reporter into mammalian cells, ABA significantly induced the reporter’s production (Figure 3C) [63]. Compared to the rapamycin system, the ABA system has two compelling advantages: first, ABA is present in many foods containing plant extracts and oils—its lack of toxicity is supported by an extensive evaluation by the Environmental Protection Agency (EPA), secondly, since the ABA signaling pathway does not exist in mammalian cells, there should be no competing endogenous binding proteins as in the rapamycin systems. To further avoid any catalysis of possible unexpected substrates by ABI1, a mutation critical for its phosphatase activity was introduced into the chimeric protein [63].

### 4.3. Induction of Target Gene by Light Sensitive Protein–Protein Interactions

Recently, two light-switchable transgene systems were developed by taking advantage of light-induced protein–protein interactions. The first one got inspiration from the molecular basis of the circadian rhythm of fungi. Vivid (VVD), a photoreceptor and light-oxygen-voltage (LOV) domain-containing protein from *Neurospora crassa*, forms a rapidly exchanging dimer upon blue-light activation [64,65]. Thus, the chimeric protein consisting of VVD and Gal4 residues 1-65 dimerizes and becomes a transcriptional activator under blue light-illumination, while the active dimer disassociates in the absence of blue light. This means that the expression of the reporter downstream of UAS can be switched on and off in a spatiotemporal manner utilizing blue light (Figure 3D). Moreover, mutagenesis optimization of VVD further reduced the background expression to a minimal level, making the system even more feasible [66]. Another light-switchable transgene system (photoactivatable (PA)-Tet-OFF/ON) exploits the *Arabidopsis thaliana*-derived blue light-responsive heterodimer formation, consisting of the cryptochrome 2 (Cry2) photoreceptor and cryptochrome-interacting basic helix-loop-helix 1 (CIB1) [67,68]. Photolyase homology region (PHR) at Cry2′s N-terminal part is the chromophore-binding domain that binds to Flavin adenine dinucleotide (FAD) by a noncovalent bond. CIB1 interacts with Cry2 in blue light-dependent manner. Thus, to make an inducible expression system, PHR was fused with the transcription activation domain of p65, and CIB1 was fused with the DNA binding, dimerization and Tetracycline-binding domains of TetR (residues 1-206) [29]. Accordingly, the reporter gene can be switched on with blue light illumination, while switching off can be achieved in two ways, either by the absence of the blue light or tetracycline addition (Figure 3E). Meanwhile, a tetracycline insensitive mutation, H100Y [69], was established to make it purely dependent on illumination. Applying the same chimeric structure, but replacing TetR with rtTA, the reporter gene can be switched on with either blue light illumination or tetracycline, and switched off either by absence of the blue light or removal of tetracycline [29]. Generally, two advantages of light-switchable transgene systems overwhelm all other systems. One is their rapid on and off cycle. Due to the nature of circadian rhythm, the two above-mentioned protein–protein interactions are dynamic, leading to a fast response and turnover. Even short pulses of light for 1–2 min are sufficient to induce luciferase expression, which has been shown to peak 1.1 h later and decline to the background level 3 h later [29]. The other advantage is its precise spatial induction. Illumination within restricted areas or cell populations can be realized with advanced illumination sources, by which the reporter expression can be selectively induced in certain cells or subcellular regions of interest. These unique features will not only greatly facilitate the future cell-cell behavior studies, but also provide vast potential for clinical gene therapy. This system is, however, not without problems, as overexposure to blue light can induce oxidative stress to cells and tissues and have especially harmful effects in sensitive tissues, such as corneal epithelium [70,71].

## 5. Tamoxifen Controlled Recombinase System

Site-specific recombinase (SSR) has been a major tool for the generation of conditional and tissue-specific knockout and knockin mice since the 1990s [72,73]. Cre from bacteriophage P1 [74] and FLP from *Saccharomyces cerevisiae* [75] are the most commonly used enzymes to orchestrate site-directed recombination. Cre and Flp recombinases recognize the 34-bp nucleotide sequence named loxP [76] or FRT [77], respectively, and precisely catalyze the excision or inversion of the gene between the two loxP or the two FRT sites [78]. In order to modulate the recombinase activity, the ligand-binding domain (LBD) of the estrogen receptor (ER) was fused with Cre [79] or Flp [80], resulting in chimeric proteins that can be activated by anti-estrogen tamoxifen or its derivative 4-OH tamoxifen (4-OH-TAM) [81]. Taking advantage of these regulatable recombinases, either single or two plasmid systems were developed to achieve inducible gene expression. The first successful case was done in mouse embryonic cells [82]. Two plasmids were transfected together. One was Cre-ER constitutive expressing plasmid, the other contained gene trap sequence flanked by LoxP, followed by β-galactosidase (LacZ) open reading frame. As a consequence, expression of LacZ could only be restored when Cre-loxP-mediated recombination was triggered and the gene trap sequence was excised [82]. By these means, the reporter gene could be induced not only in undifferentiated embryonic stem cells and embryoid bodies, but also in all tissues of a 10-day-old chimeric fetus or specific differentiated adult tissues [83]. In another example, to induce enhanced green fluorescent protein (EGFP) expression in baby hamster kidney (BHK) cells and to simplify the plasmid construction, Cre-ER cDNA flanked by LoxP sites were inserted between phosphoglycerate kinase (PGK) promoter and EGFP encoding sequence. In this system, Cre-ER functions as a gene trap to block the transcription of EGFP without 4-OH-TAM. Ignition of recombinase activity by 4-OH-TAM melts off the Cre-ER cassette and restores EGFP expression driven by PGK promoter [81]. To exclude the effect exerted by endogenous steroids, three distinct ERs are mostly exploited: (1) mouse ERTM with a G525R mutation [84], (2) human ERT with G521R mutation [80] and (3) human ERT2 containing three mutations G400V/M543/L544A [85].

## 6. Riboswitch-Regulatable Expression System

All the above-mentioned systems are dependent on exogenous proteins. They have two inborn drawbacks. One is that exogenous proteins hold the risk of inducing immunogenic reactions in vivo. The other is that construction and transfection of large-sized plasmids required for these systems can burden the host cells. To overcome these shortcomings, a riboswitch-regulatable expression system has emerged and has been further developed to achieve inducible gene expression/knockdown in mammalian cells since 2009 [86]. This system takes advantage of bacteria-derived RNA aptamers linked with hammerhead ribozymes (aptazymes). Aptamer acts as a molecular sensor and transducer for the whole apparatus, while ribozyme responds to the signal with conformation change and mRNA cleavage. For example, Gram-positive bacteria’s aptazyme can directly sense excessive glucosamine-6-phosphate (GlcN6P) and cleave mRNA of the *glms* gene, whose protein product is an exzyme that converts fructose-6-phosphate (Fru6P) and glutamine to GlcN6P [87]. Most natural aptazymes don’t function in mammalian cells. To date, only a few synthetic aptazymes have been screened out and applied for efficient control of gene expression in mammalian cells [86,88,89,90]. These aptazymes, responding to tetracycline, theophylline, guanine, etc. were engineered to both knock down and overexpress the gene of interest. In a recent review written by the pioneer of riboswitch regulatable expression system, Yohei Yokobayashi summarized extensively the diversity of the regulatory mechanisms harnessed by riboswitch [91]. Therefore, we will not cover this topic in more detail here. Although the potential of riboswitch is inspiring, most of the aptazymes do not make a comparable induction fold as the other systems, as described in Table 2. Moreover, leaky expression and basal knockdown are common for this system. Therefore, further optimization and development are expected to be made in the future.

## 7. Closing Remarks and Future Perspectives

### 7.1. How to Choose the Right System for the Experiment

Since the 1980s, tremendous progress has been made to control gene expression in a temporal and tunable manner. It has mainly branched in three directions: one is to manipulate chimeric transcription factor’s activity, the second is to modulate recombinase’s activity, and the third is to regulate ribozyme’s activity by an aptamer. By virtue of their nature, most systems in the first and third category are reversible but lack spatial regulation, while the second category is irreversible. Therefore, the majority of the efforts have been put in optimization and development of the systems utilizing the chimeric transcription factors. In this case, the chimeric transcription factor often consists of either the endogenous nuclear hormone receptor, or engineered operator, or a counter partner of protein–protein interaction, together with an activation domain fusion protein whose association with the former part can be triggered by various inducers.

To date, tetracycline/cumate-controlled operator systems are preferred for routine inducible-expression/knockdown experiments due to the easiness of their handling, high efficiency, and negligible side effects. However, different options of configurations, promoters and activating domains still complicate the selection. The Tet-on and the reverse activator configuration of cumate system are usually prioritized due to their negligible leakiness. The Tet-off configuration and activator configuration of cumate system are preferably selected when experiments need to avoid the presence of tetracycline and cumate in the culture medium. Promoters and activating domains determine the strength of the induction. In the case of induction of a specific gene, minimal constitutive promoters are employed. So accordingly, human elongation factor 1α promoter (EF1A) and CMV promoter are recommended for higher induction, while human Ubiquitin C promoter (UBC) and PGK promoter are recommended for medium or lower induction [92]. In the case of induction of shRNA, there is no dramatic difference between the use of H1 or U6 promoter (REFERENCE MISSING). Regarding activating domains, parental and tandemly reiterated VP16 and p65 are mostly used. Of these, p65 was shown to be a stronger transcription activator compared to VP16 [93]. Thus, different combinations of promoter and activating domains can be used to achieve inductions with differing extents, depending on experimental requirements. Maximum induction of a certain gene by tetracycline/cumate-controlled operator systems can be up to 100–1000 folds when the endogenous protein is lowly expressed [94].

For more complicated induction requirements, such as repetitive and spatial inductions, light-switchable systems provide better options. With these systems, the maximum induction rate of the gene of interest ranges from 50 to 100 folds [29], making them almost comparable with tetracycline/cumate-controlled operator systems.

To further compare the reviewed induction systems, an overview of the cons and pros of each system is displayed in Table 2. Not one of the systems is perfect in every aspect, as the most suitable should be the one with a maximum advantage critical for experimental purpose and with minimum compromise of other features. The availability of the different inducible-expression systems are presented in Table 3.

### 7.2. Future Perspectives

One important lesson we can learn from the development of regulatable expression systems is that the more we know about how genes are regulated in different organisms, the better we can take advantage of this knowledge and harness it for research. Even though the tools we have now are satisfactory for many purposes, more advanced regulatable expression systems could still expand our research possibilities. e.g., for the light-switchable system, chimeric transcription factor responding to lights of different wavelengths could be developed by functional screening after random and/or designed mutagenesis. For the protein–protein interaction-based chimeric transcription factor, a selection of a pair of proteins that can be associated and disassociated by different drugs or stimuli in a sequential manner could make the induction even more precise and controlled.

## Figures and Tables

**Table 1:** Bacterial operators other than LacO, TetO and CuO.

Regulator Protein	Origin	Inducer	Association or Disassociation	Reference
AlcR	*A. nidulans*	Acetaldehyde	Association	[95]
ArgR	*C. pneumoniae*	l-Arginine	Association	[96]
BirA	*E. coli*	Biotinyl-AMP	Association	[97]
EthR	*M. tuberculosis*	2-Phenylethyl butyrate	Dissociation	[98]
HdnoR	*A. nicotinovorans*	6-Hydroxy nicotine	Dissociation	[99]
HucR	*D. radiodurans*	Uric acid	Dissociation	[100]
MphR(A)	*E. coli*	Macrolides	Dissociation	[101]
PIP	*S. pristinaespiralis*	Streptogramins	Dissociation	[26]
Rex	*S. coelicolor*	NADH	Dissociation	[102]
RheA	*S. albus*	Heat	Dissociation	[103]
ScbR	*S. coelicolor*	SCB1	Dissociation	[104]
TraR	*A. tumefaciens*	3-oxo-C8-HSL	Association	[105]
TtgR	*P. putida*	Phloretin	Dissociation	[106]

**Table 2:** Brief comparison of reviewed induction systems (listed according to inducer).

Inducer	Reversability	Maximum Induction	Overexpression	Knockdown or Knockout	Spatial Regulation
Tetracycline	Yes	100-1000X	Yes	Yes	No
Cumate	Yes	100-1000X	Yes	Yes	No
Rapamycin	Yes	100-1000X	Yes	NT	No
FKCsA	Yes	100-1000X	Yes	NT	No
ABA	Yes	100-1000X	Yes	NT	No
Tamoxifen	No	30-50X	Yes	Yes	No
Blue light	Yes	50-100X	Yes	NT	Yes
riboswitch	Yes	5-9X	Yes	Yes	No

NT: not tested, ABA: abscisic acid.

**Table 3:** Commercially available and Addgene-stored plasmids (those with modified tags are not included).

Plasmid Name	Type of System	Purpose	Company/Addgene	Resource NO.
pTRE3G	Operon	Overexpression	Takara	631167, 631168
pCMV-Tet3G	Operator	Overexpression	Takara	631168
pEF1a-Tet3G	Operator	Overexpression	Takara	631167
pLVXTRE3G	Operon & lentiviral	Overexpression	Takara	631187, 631363
pcDNA™4/TO	Operon	Overexpression	Thermo Fisher	K1020-01
pcDNA™5/TO	Operon	Overexpression	Thermo Fisher	V103320
pcDNA6/TR	Operator	Overexpression	Thermo Fisher	V103320
pEGSH	Fly heat shock promoter driven casssette	Overexpression	Agilent	217461
pERV3	Fly ecydysone receptor	Overexpression	Agilent	217460
pF12A RM Flexi^®^	Operon	Overexpression	Promega	C9431
pF12K RM Flexi^®^	Operon	Overexpression	Promega	C9441
pCDH-CuO-MCS-IRES-GFP-EF1α-CymR-T2A-Puro	Operon & lentiviral	Overexpression	SBI	QM812B-1
PB-Cuo-shMCS-IRES-GFP-EF1α-CymR-Puro	Operon	Knockdown	SBI	PBQMSH812A-1
Tet-pLKO-neo/puro	Operon	Knockdown	Addgene	21915, 21916 [107]
pCW57.1	Operon	Overexpression	Addgene	41393
pLVUT-tTR-KRAB	Operator & lentiviral	Knockdown	Addgene	11651 [108]
pSLIK-neo	Operon & lentiviral	Knockdown	Addgene	25735 [109]
pPRIME-TET-GFP-FF3	Operon & lentiviral	Knockdown	Addgene	11662 [110]
pTet-IRES-EGFP	Operon	Overexpression	Addgene	64238 [111]
pCAG-CreERT2	Recombinase	Overexpression	Addgene	14797 [112]
